# Peripheral Blood Lymphocyte Percentage May Predict Chemotolerance and Survival in Patients with Advanced Pancreatic Cancer. Association between Adaptive Immunity and Nutritional State

**DOI:** 10.3390/curroncol28050285

**Published:** 2021-08-26

**Authors:** Roberto Aquilani, Silvia Brugnatelli, Roberto Maestri, Federica Boschi, Beatrice Filippi, Lorenzo Perrone, Annalisa Barbieri, Daniela Buonocore, Maurizia Dossena, Manuela Verri

**Affiliations:** 1 Department of Biology and Biotechnology “Lazzaro Spallanzani”, University of Pavia, 27100 Pavia, Italy; dottore.aquilani@gmail.com (R.A.); daniela.buonocore@unipv.it (D.B.); maurizia.dossena@unipv.it (M.D.); manuela.verri@unipv.it (M.V.); 2 Medical Oncology Division, Fondazione IRCCS, Policlinico San Matteo, 27100 Pavia, Italy; S.Brugnatelli@smatteo.pv.it (S.B.); beatrice.filippi02@universitadipavia.it (B.F.); lorenzo.perrone01@universitadipavia.it (L.P.); 3 Department of Biomedical Engineering of the Montescano Institute, Istituti Clinici Scientifici Maugeri IRCCS, 27040 Montescano, Italy; roberto.maestri@icsmaugeri.it; 4 Department of Drug Sciences, University of Pavia, 27100 Pavia, Italy; annalisa.barbieri@unipv.it

**Keywords:** advanced pancreatic cancer, impaired adaptive immune response, chemotolerance, survival, nutritional status

## Abstract

**Simple Summary:**

Alterations of the immune system that consist of induced inflammation and reduction in blood lymphocytes are a major factor contributing to cancer progression in patients with pancreatic carcinoma (PC). Identification of the percentage of lymphocytes in the Total number of White Blood Cells (L% TWBC) that could be associated with chemotolerance and survival time may be important for predicting treatment efficacy. The aim of this retrospective study was to highlight the best value of L% for predicting chemotolerance (n° of cycles tolerated) and survival beyond 12 months from diagnosis. The study found that L ≥ 29.7% TWBC compared to L < 29.7% predicted chemotolerance (*p* < 0.0001) and survival at every time point of follow-up: 6 (*p* = 0.04), 12 (*p* = 0.0003) and 18 months (*p* = 0.004) after diagnosis. Simple, rapid, routine laboratory data can be useful to predict treatment tolerance and efficacy in PC patients.

**Abstract:**

Pancreatic Carcinoma (PC) cells have the ability to induce patient immunosuppression and to escape immunosurveillance. Low circulating lymphocytes are associated with an advanced stage of PC and reduced survival. Blood lymphocytes expressed as a percentage of Total White Blood Cells (L% TWBC) could predict chemotolerance (n° of tolerated cycles), survival time and Body Weight (BW) more effectively than lymphocytes expressed as an absolute value (L_AB_ > 1500 n°/mm^3^) or lymphocytes >22%, which is the lowest limit of normal values in our laboratory. Forty-one patients with advanced PC, treated with chemotherapy, were selected for this observational retrospective study. Patients were evaluated at baseline (pre-chemotherapy), and at 6, 12 and 18 months, respectively, after diagnosis of PC. The study found L ≥ 29.7% to be a better predictor of survival (COX model, using age, sex, BW, serum creatinine, bilirubin and lymphocytes as covariates), chemotolerance (r = +0.50, *p* = 0.001) and BW (r = +0.35, *p* = 0.027) than L_AB_ > 1500 or L > 22%. BW did not significantly correlate with chemotolerance or survival. The preliminary results of this study suggest that L ≥ 29.7% is more effective than L_AB_ > 1500 or L > 22% at predicting chemotolerance, survival time and nutritional status. A possible impact of nutritional status on chemotherapy and survival seems to be lymphocyte-mediated given the association between BW and L%. This study may serve as the basis for future research to explore whether nutritional interventions can improve lymphopenia, and if so, how this may be possible.

## 1. Introduction

Pancreatic cancer (PC) is a highly aggressive malignancy with a poor prognosis for patients. PC is the fourth most important cause of death worldwide [1]. Most PC patients die within the first year after diagnosis [2,3,4] and the 5-year survival rate is less than 10% [5,6]. In our country (Italy), the survival rate is 8.1% at 5 years and 3% at 10 years after diagnosis [7]. Surgery is the most important therapeutic option for localized PC. Despite the progress made in recent decades, the recurrence rate five years after resection is 80%, and the survival rate is 22% [8,9].

Besides genetic alterations in cancer cells—responsible for tumor cell growth and chemoresistance—abnormal host immunity is a major factor contributing to disappointing results of PC treatment. Cancer itself induces dysfunctions in the host’s immune system by causing immunosuppression [10,11,12,13,14,15]. The host thus loses his/her capacity to fight/eliminate cancer cells. Indeed, both cancer development and progression are associated with the progressive deterioration of immunosurveillance [12,15]. Contrast surgery [16] and chemoradiotherapy [16] improve the immune dysfunction in PC patients.

Several investigations found that alterations in immune responses in PC patients (and in patients with other types of cancer cells) consist in dysfunctions of both innate and adaptive immune systems.

Regarding the innate immune response, inflammation—the first-line response to cancer cell antigens—plays a crucial role in the development and progression of cancer [17,18,19]. Indeed, the surrogates of inflammation, such as C-reactive protein (CRP) and the ratios between circulating neutrophils and platelets on one hand and lymphocytes on the other hand, were found to have prognostic significance in PC patients undergoing surgical treatment [20,21,22,23]. Recently, the systemic immune-inflammation index (i.e., platelet count x neutrophil/lymphocyte ratio) was found to independently predict survival and recurrence in PC patients treated with surgery [24].

Impairments of adaptive immune responses greatly influence both PC development and progression. The reduction in total lymphocyte count is common in PC [25] and is responsible for reducing immunosurveillance and dissemination of circulating cancer cells [17].

Blood lymphopenia has been shown to be the main immunological alteration in metastatic PC [26]. Moreover, impaired adaptive immune responses seem more important than innate immunity as negative prognostic factors given that a recent investigation reported that, even though the neutrophil to lymphocyte ratio was a predictor of patient survival after resection of early-stage pancreatic cancer [27], the lymphocyte count alone shows the same survival curve as the ratio, whereas neutrophil count alone was not a significant predictor. This could mean that lymphocyte counts alone have a prognostic value in patients resected for early-stage pancreatic cancer.

The comprehension of the role played by lymphocytes in cancer development/destruction is an unmet need. To the best of our knowledge, no study has documented the level of circulating lymphocytes as a potential predictor of chemotolerance and clinical outcomes in patients with advanced PC. We speculated that circulating lymphocytes expressed as a percentage (L%) of Total White Blood Cells (TWBC) could predict chemotolerance and clinical outcomes better than lymphocytes expressed in absolute values (L_AB_). Indeed, L% would imply a balance between the innate and adaptive immune responses, which is not captured by L_AB_. Therefore, the primary goal of the current observational retrospective study was to explore whether L% could predict chemotolerance and clinical outcomes, and if so, what the optimal L% value might be.

The second goal was to investigate whether body weight (BW)—an index of good health—was associated with L% more than with L, chemotolerance and clinical outcomes. As nutrition is crucial for lymphocyte proliferation and metabolic activities [28,29], we supposed that body weight might improve L% which, in turn, might have a positive impact on both chemotolerance and clinical outcomes.

The first objective of the study was to examine L% as a predictor of chemotolerance and clinical outcomes. We hypothesized that L% would be superior to L_AB_ in predicting chemotolerance, clinical outcomes and survival because L% would better imply a balance between innate and adaptive immunity. Moreover, we tried to identify the cutoff value of L% that could predict chemotolerance and clinical outcomes.

The second objective of the study was to document the relationship between body weight (BW)—an indicator of good health—and circulating neutrophil counts (N_AB_), L_AB_, neutrophil/lymphocyte ratio (N/L), chemotolerance and survival.

The ultimate purpose of our investigation was to highlight possible bases for future research into the potential effects of nutritional interventions to correct lymphopenia, ensure chemotolerance and improve clinical outcomes of patients with advanced PC.

## 2. Materials and Methods

### 2.1. Patients

This observational retrospective study was approved by the Ethical Committee of Policlinico S. Matteo (Pavia, Italy) (P-20210006621, 4 June 2021). Written informed consent was obtained from each patient after accepting chemotherapy following our standard protocol.

The medical records of patients diagnosed with stage IV PC, admitted from 1 January 2018 to 31 December 2020 were extracted from the database in the Department of Oncology.

The inclusion criteria were the following: (1) histological/cytological diagnosis of non-resectable exocrine pancreatic cancer (any T, any N M1 and T4) carried out between 1 January 2018 and 31 December 2020; (2) age > 18 years; (3) Karnofsky Index ≥ 70; (4) hemoglobin concentrations > 9 g/dL; circulating neutrophils and platelet counts > 1500/mm^3^ and > 100,000/mm^3^, respectively; serum creatinine < 1.5 mg/dL; total bilirubin < 1.5 times the upper limits of the our laboratory; serum albumin > 3 g/dL; (5) patients who underwent first-line chemotherapy following the AIOM 2020 guidelines (Folfirinox, Nab paclitaxel—Gemcitabin, PEX-G, Gemcitabin as the only therapy); (6) patients with biohumoral variables including serum levels of lactate dehydrogenase (LDH) enzyme. The exclusion criteria were the following: (1) patients with potentially resectable cancer; (2) patients with insula cell cancer; (3) patients < 18 years; (4) patients on adjuvant anticancer drugs 6 months before the diagnosis of advanced disease (tumor recurrence); (5) patients with clinically significant cardiovascular disease and hemodynamic instability; (6) presence of autoimmune diseases or active collagenopathies; (7) diabetes mellitus with poor metabolic control; (8) patients who had not completed at least one cycle of chemotherapy.

### 2.2. Materials and Procedures

Demographic data, anthropometric measurements, laboratory data, number of chemotherapy cycles provided, and oncologic data, including Computed Tomography examination (CT), were recorded. Patients were subsequently categorized for levels of circulating lymphocytes (L). Lymphopenia was defined both in absolute values (L_AB_) (< 1500/mm^3^) and in percentages of total blood white cells (TWBC) (L < 22%). L_AB_ 1500 and L 22% are the normal lower limits of lymphocytes in our laboratory.

Moreover, the patients were stratified for the occurrence of unfavorable outcomes (Disease Progression: DP, death) or favorable outcomes (Stable Disease: SD, Partial/Complete Response: PR, to chemotherapy).

The patients were followed up until death or admission to a palliative care setting.

### 2.3. Definitions

Overall Survival (OS) was calculated from the beginning of the first cycle of first-line chemotherapy until the date of the patient’s death or last follow-up. Progression-Free Survival (PFS) was calculated from the beginning of the first cycle of first-line chemotherapy until the date of cancer progression, radiologically and/or clinically documented.

Following the standard treatment protocol used in the Oncology Department, patients received chemotherapy until disease progression and/or unacceptable toxicity.

Chemotolerance was defined as patients’ capacity to tolerate all the scheduled cycles of chemotherapy. Patients for whom this was not the case were defined as having poor tolerance.

## 3. Results

Patients’ clinical and biohumoral variables, n° of tolerated cycles are described in Table 1 and Table 2. They had normal body weight (body mass index, BMI) (Table 1), hepatic dysfunction (high levels of serum aminotranspherases, alkaline phosphatase, ɣ-glutamyl transpeptidase), poor control of glucose metabolism (hyperglycemia), increased rate of body cell anaerobic metabolism (high serum concentrations of acid Lactic Dehydrogenase (LDH) enzyme) and high plasma tumor markers (Table 2).

### 3.1. Lymphopenia and Relationship with Chemotolerance and Clinical Outcomes

Lymphopenia expressed as L_AB_ was found in 56.4% of patients (Table 3a). Circulating neutrophil counts were similar in subjects with and without lymphopenia. Patients with lymphopenia exhibited a higher innate immune response (N/L ratio) than patients with normal circulating L_AB_ (*p* = 0.017) (Table 3a). No significant differences were found in the chemotolerance between lymphopenic and non-lymphopenic patients (*p* = 0.53).

Lymphopenia, expressed as a percentage of total white blood cells (TWBC) (L < 22%) (Table 3b), was found in 53.8% of the patients, similar to that expressed as L_AB_. In patients with L < 22%, neutrophils in absolute values and as a percentage of TWBC as well as the N/L ratio were significantly higher than in patients with L > 22% (Table 3b).

Of note, L_AB_ < 1500 and L% < 22% were related (*p* chi-sq = 0.007), but 13% of the patients had L_AB_ > 1500 and L% < 22% and 15% had L_AB_ < 1500 and L% > 22%.

No significant difference in chemotolerance was observed between patients with L% < 22% and with L% > 22% (*p* = 0.15).

**Figure 1:**
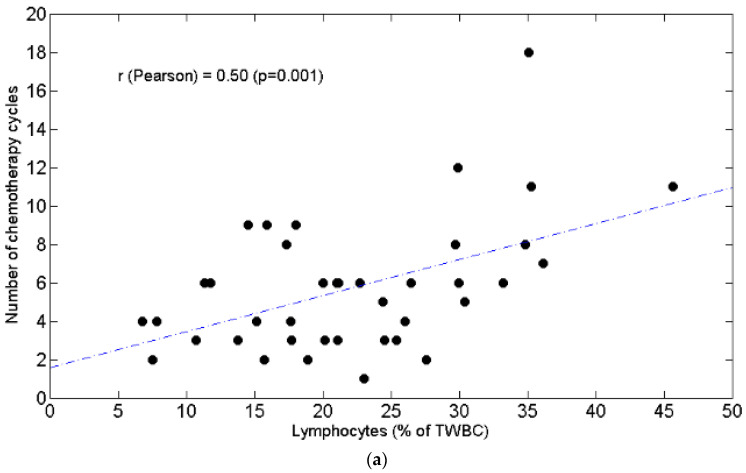

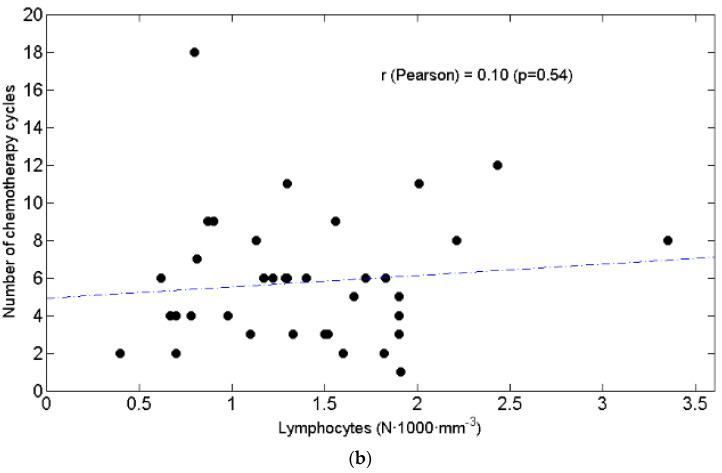
Scatter plots of the number of chemotherapy cycles (y axis) as a function of circulating lymphocytes (x axis) expressed as percentage of total white blood cells (**a**) and as absolute value (**b**). The value of Pearson’s correlation coefficient and corresponding *p* value are also reported. TWBC: total white blood cells.

Correlation analysis (Pearson *r*) revealed that chemotolerance was positively associated with L% (Figure 1a, r = 0.5, *p* = 0.001) but not with L_AB_ (Figure 1b, r = 0.1, *p* = 0.54).

Patients who experienced or did not experience a poor outcome had similar values of L_AB_ (1.4 ± 0.5 vs. 1.4 ± 0.7, *p* = 0.85) and showed a tendency towards lower values of L%, but statistical significance was not reached (19.7 ± 6.7 vs. 24.2 ± 10.3, *p* = 0.12).

To determine the value of L% with the best discriminant power (with regards to a poor outcome), Receiver Operating Characteristic (ROC) curves were built and the Area Under the Curve (AUC) was computed. The optimum value for L% was 29.7% (Sensitivity = 0.94, Specificity= 0.43). This value was also the upper quartile of L% distribution.

Logistic regression analysis revealed that patients with L% < 29.7% had a 13-fold higher risk for poor outcomes (95% CI: 1.4–11.1, *p* = 0.023). This strong association was also confirmed when results were adjusted for age, sex, plasma total bilirubin, serum creatinine, ɣ-glutamyl transpeptidase and estimated glomerular filtration rate (odds ratio = 25, *p* = 0.007).

Chemotolerance was lower in patients with L < 29.7% than in patients with L ≥ 29.7% (n° cycles 4.6 ± 2.3 vs. 9.2 ± 3.9, *p* < 0.0001) (Table 3c).

Table 3d. reports absolute neutrophils, neutrophils%, absolute lymphocytes, lymphocytes%, neutrophils/lymphocytes ratio, and chemotherapy cycles in patients after stratification for their response to first-line chemotherapy (PR partial response, SD stable disease, PD progressive disease). The table indicates the association between clinical outcomes and circulating lymphocytes. Total lymphocytes were similar among the three groups (*p* = 0.53) whereas lymphocytes% were higher in SD group than in PR one (27.4% ± −3.5 vs. 22.17% ± −2.1%, *p* < 0.001) and in PD patients (19.68% ± −1.63%, *p* < 0.001). Moreover, lymphocytes% resulted significantly higher in PD group than in PR group (*p* = 0.02).

### 3.2. L ≥ 29.7% as a Predictor of Survival

**Figure 2:**
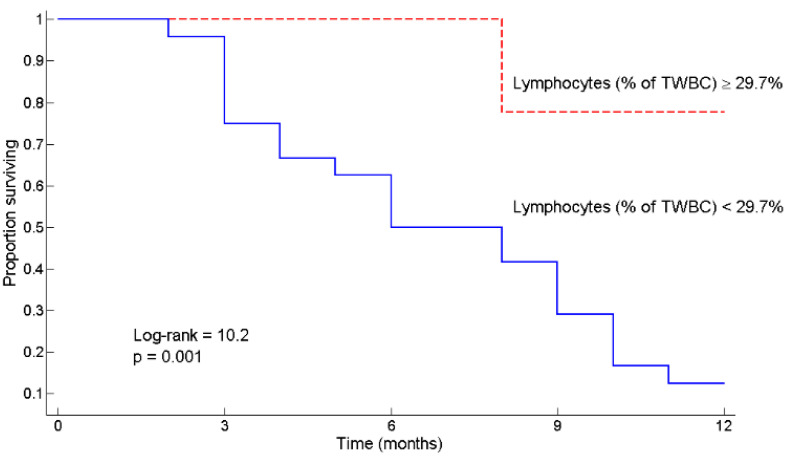
Kaplan–Meier survival curves according to lymphocytes expressed as percentage of total white blood cells ≥ 29.7% and < 29.7%. The 12-month mortality rate varied from 22% for patients with lymphocytes ≥ 29.7% of total white blood cells to 87% for patients with lymphocytes < 29.7% of total white blood cells. TWBC: total white blood cells.

Patients were first divided into two groups using L < 29.7% as the threshold, and then one-year survival curves were estimated by the Kaplan–Meier method and compared by the log-rank test. L ≥ 29.7% predicted patient survival at 12 months from diagnosis of pancreatic cancer (Figure 2) (Log-rank = 10.2, *p* = 0.001). Indeed, the mortality rate was higher in patients with L < 29.7% at any time point: at 6 months it was 32% vs. 0 (*p* = 0.04), at 12 months it was 87 vs. 22% (*p* = 0.0008), and at 18 months it was 96 vs. 37% (*p* = 0.0018).

### 3.3. Relationships between BW and Lymphocytes, Neutrophils, N/L Ratio, Chemotolerance and Clinical Outcomes

**Figure 3:**
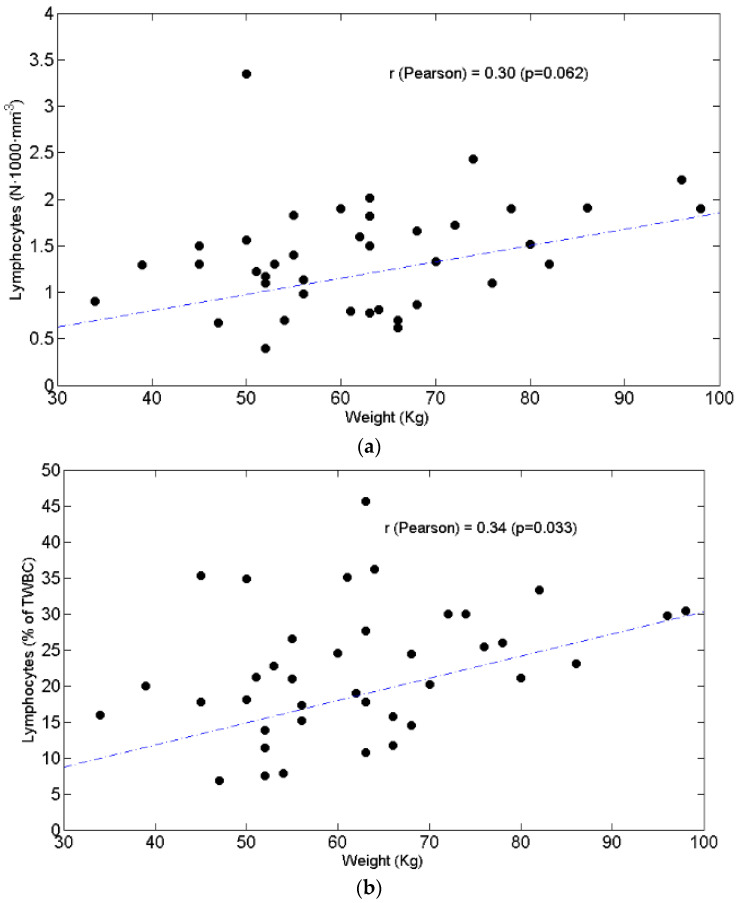
Scatter plots of the circulating lymphocytes (y axis) as a function of weight (x axis). Lymphocytes are expressed as absolute value (**a**) and as percentage of total white blood cells (**b**). The value of Pearson’s correlation coefficient and corresponding *p* value are also reported. TWBC: total white blood cells.

BW did not significantly correlate with L_AB_ (Figure 3a) (r = +0.30, *p* = 0.062) but correlated L% (Figure 3b) (r = +0.34, *p* = 0.033).

**Figure 4:**
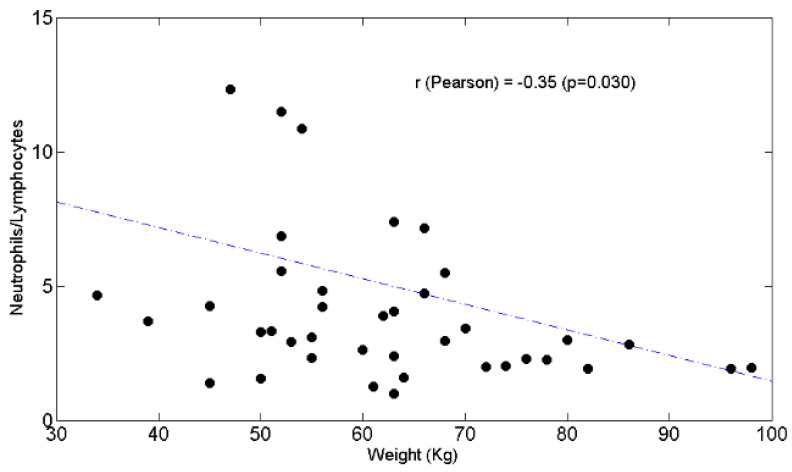
Scatter plot of the neutrophils/lymphocytes ratio (y axis) as a function of weight (x axis). The value of Pearson’s correlation coefficient and corresponding *p* value are also reported.

**Figure 5:**
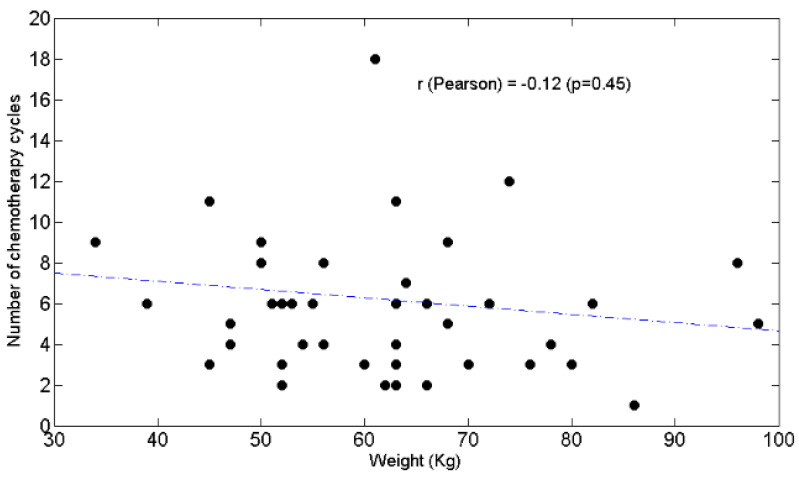
The number of chemotherapy cycles is not significantly linked to body weight (Kg). Scatter plot of the number of chemotherapy cycles (y axis) as a function of weight (x axis). The value of Pearson’s correlation coefficient and corresponding *p* value are also reported.

**Figure 6:**
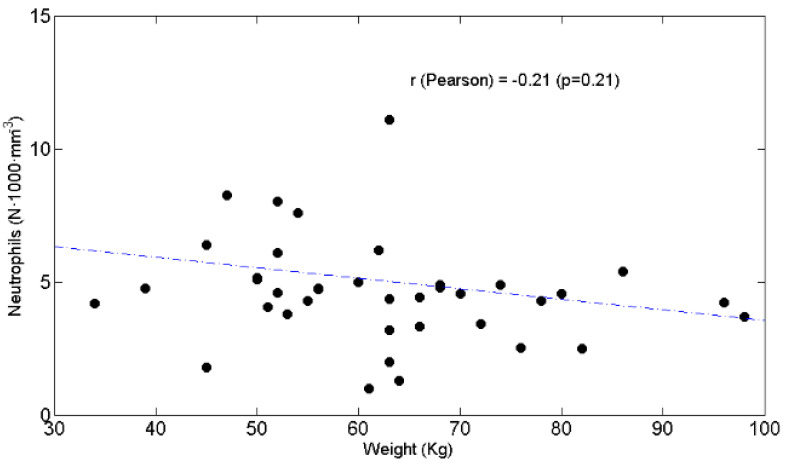
Scatter plot of the circulating neutrophils expressed as absolute value (y axis) as a function of weight (x axis). The value of Pearson’s correlation coefficient and corresponding *p* value are also reported.

BW was negatively associated with the N/L ratio (Figure 4) (r = −0.35, *p* = 0.03) but not with chemotolerance (Figure 5) (r = −0.12, *p* = 0.45) or neutrophil counts (Figure 6) (r = −0.21, *p* = 0.2).

**Figure 7:**
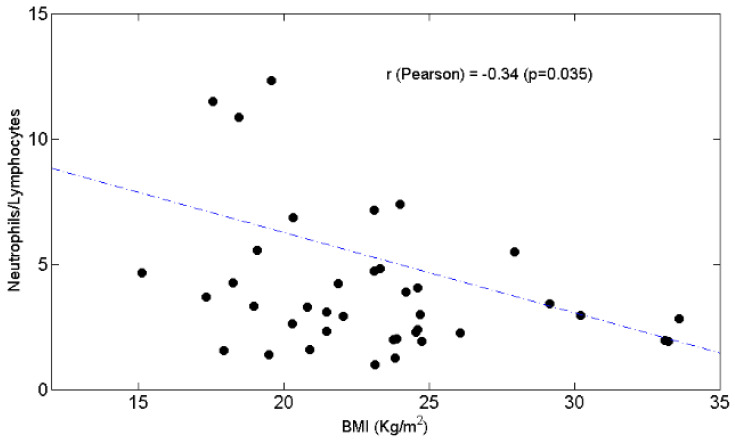
Scatter plot of the neutrophils/lymphocytes ratio (y axis) as a function of BMI (Body Mass Index) (x axis). The value of Pearson’s correlation coefficient and corresponding *p* value are also reported.

BMI was negatively correlated with N/L ratio (Figure 7) (r= −0.34, *p*= 0.035) but not with absolute neutrophils (r = −0.12, *p* = 0.45), L%(r = +0.26, *p* = 0.11) and L_AB_ (r = +0.27, *p* = 0.09), number of chemotherapy cycles (r = −0.09, *p* = 0.56).

Figure 1. The number of chemotherapy cycles is positively associated with circulating lymphocytes expressed as percentage of Total White Blood Cells (Figure 1a). The number of chemotherapy cycles is not significantly linked to circulating absolute lymphocytes (Figure 1b).

Figure 2. Mortality rate varies in relation to circulating lymphocytes expressed as percentage of Total White Blood Cells.

Figure 3. Circulating absolute lymphocytes are not significantly associated with body weight (Kg) (Figure 3a). Circulating lymphocytes as percentage of Total White Blood Cells are significantly linked to body weight (Kg) (Figure 3b).

Figure 4. Blood neutrophils/lymphocytes ratio is negatively correlated with body weight (Kg).

Figure 5. The number of chemotherapy cycles is not significantly linked to body weight (Kg).

Figure 6. Circulating absolute neutrophils are not significantly correlated with body weight (Kg).

Figure 7. Blood neutrophils/lymphocytes ratio is inversely associated with Body Mass Index (kg/m^2^).

## 4. Discussion

The study shows that in patients with advanced PC, peripheral blood L ≥ 29.7% represented an independent positive predictor of both chemotolerance and clinical outcomes. Moreover, the results show that BW was positively associated with the percentage of circulating lymphocytes and negatively associated with the prevalence of innate immune response (a high N/L ratio) as BW did not correlate with chemotolerance or clinical outcomes. Lymphopenia expressed both as L_AB_ < 1500 and L < 22%, failed to identify patients with chemointolerance, unfavorable clinical outcomes or association with BW.

### 4.1. Patients’ Characteristics

The patients did not have liver tumor infiltration. The hepatic dysfunction could be due to liver lesions that were not identified at the time of diagnosis [30]. The lack of glucose metabolism control (hyperglycemia) is a common condition of PC and accompanies 85% PC patients [31]. Hyperglycemia is caused by endocrine pancreatic insufficiency. In addition, a potential factor contributing to hyperglycemia might be increased neoglycogenesis activity following an excess of muscle amino acid release [32]. Hyperglycemia induces an accumulation of hypoxia-inducible factor-1 alpha (HIF-1α) that mediates the increase in both glucose breakdown through the anaerobic pathway and the activity of cellular lactate dehydrogenase (LDH). Hyperglycemia and high LDH levels, which were both found in the study, are signals of risk for tumor progression [31].

Patients’ innate immune response was slightly higher than their adaptive immune response (a high N/L ratio). This imbalance between the two arms of immunity was due to persistent inflammation, an important factor for both the development and progression of cancer [24,27].

### 4.2. Lymphopenia, Chemotolerance, Clinical Outcomes and BW

In our investigation, the prevalent lymphopenia is in line with the findings of previous studies [26] carried out on PC patients. Lymphopenia (L < 1200 n°/mm^3^) in patients with advanced stages of PC [26] contributed to the prediction of adverse outcomes. In our study, this was not the case for patients with lymphopenia L_AB_ 1000 ± 300 n°/mm^3^). The importance of lymphocytes as a prognostic predictor was also reported in subjects who underwent surgical treatment for early stage pancreatic ductal adenocarcinoma [27]. In this study, lymphocytes alone surrogated the predictivity of the prognostic value of the N/L ratio. The results of the current investigation confirm the prognostic value of circulating lymphocytes and add the information that L ≥ 29.7% TWBC was the best cutoff value to predict both chemotolerance and clinical outcomes.

An interesting dilemma is why L ≥29.7% was shown to potentially predict patients’ prognosis more than L_AB_ or L < 22%. We postulate that L 29.7% may imply a better balance between adaptive and innate immune responses: high L% would suggest that the adaptive immune system activity is higher than the innate immune system activity. L 29.7% would therefore indicate the minimal value, signaling a shift in the immune system response towards higher adaptive immunity. This is indicative of an efficient patient immunosurveillance and a better control of PC progression. This may explain our study results. Indeed, a normal adaptive immune system (L ≥ 29.7%) might have favored or enhanced a reduction in immunosuppressive cytokines induced by chemotherapy. On the other hand, a higher innate immune response than adaptive immune response (high N/L ratio) was reported to predict a poor prognosis in various cancer types [23,33,34,35].

Moreover, our study showed that high adaptive immune activity was probably associated with a reduction in glucose anaerobic metabolism and lactate formation. Indeed, patients with L ≥ 29.7% had lower serum LDH values. Given the retrospective nature of this study, no causal relationship can be set between L ≥ 29,7% and serum LDH concentrations. However, inflammation, belonging to the innate immunity arm, has been showed to reduce the activity of glucose aerobic metabolism while favoring the anaerobic pathway of glucose breakdown [36].

The capacity of L ≥ 29.7% to predict chemotolerance and clinical outcomes suggests the ability to counteract the downregulation of the host’s adaptive immunity exerted by pancreatic tumors, responsible for the escape from immunosurveillance [26]. Indeed, pancreatic cancer cells reduce total lymphocyte counts and T helper cells [26,37,38,39,40].

At any time point in patient follow-up, subjects with L < 29.7% tolerated far fewer cycles of chemotherapy than those with L ≥ 29.7%.

The fact that both BW and BMI negatively correlate with N/L ratio but not with absolute neutrophils and BW alone with L% may suggest that a good nutrition may positively influence immune response by improving adaptive immune cells and favouring the shift of innate to adaptive immunity.

### 4.3. Potential Mechanisms Linking L ≥ 29.7% to Chemotolerance and Clinical Outcomes

We postulate that the absence of infection occurrence (data not shown) and good general health might have contributed to patients’ tolerance and favorable outcomes. Indeed, infection immediately causes inflammation and high innate immune activity.

The fact that BW positively correlated with L% and negatively with N/L ratio, and the lack of any significant direct relationship with chemotolerance and clinical outcomes mean that normal/good nutrition is associated with a prevalent adaptive immune function. On the other hand, it is well documented that nutrition plays a crucial role for the immune system. Firstly, in cancer patients, particularly those with PC, malnutrition causes immune dysfunction [26] and reduces T cell capacity to consume nutrients [41]. Secondly, the metabolism of T cells is strongly dependent on nutrition status and nutrient intakes, both at rest and under activation [41], in order to ensure both proliferation and function activities. Thirdly, the type of nutrients consumed by T cells changes not only in relation to the T cell subsets but also to their metabolic modifications after activation [29].

The complex metabolic activities of T lymphocytes primed by nutrition might explain why BW was not directly related to chemotolerance but was L%-mediated.

In addition to nutrition, a factor that might explain the predictive value of L ≥ 29.7% for chemotolerance and clinical outcomes is the anatomical and functional integrity of the orogastrointestinal tract. The digestive tract of lymphopenic patients may be more exposed to anticancer agent toxicity given that more than 50% of body adaptive immunity resides in the small intestine. Altered gut immunity may bring about a number of adverse effects including dysbiosis, damage to the intestinal anatomical and functional integrity and increased risk of local and systemic inflammation, the development of mucositis, nausea, vomiting, abdominal discomfort, bloating and diarrhea, all of which are factors that reduce patients’ chemotolerance. In support of the above, gut dysbiosis [42] may have an impact on PC progression because it influences immunosurveillance [43].

Therefore, adequate adaptive immune activity is an important factor that potentially reduces the incidence of adverse digestive problems leading to chemotherapy discontinuation, interruption or reduction in scheduled drug doses.

The maintenance of a normal gastrointestinal immune function may contribute to accounting for the association of L% with chemotolerance.

### 4.4. Strengths and Limitations

The current study provides very simple and immediate information in clinical practice to predict chemotolerance and clinical outcomes of patients with advanced PC.

The physician may consider a patient with circulating lymphocytes L ≥ 29.7% as having an improved balance between the adaptive and innate immune responses; this is a very important concept in oncological clinical practice because successful chemotherapy and other treatments rely on the recovery of anticancer immune responses [44].

This study has several limitations that require further investigation. Our research was carried out in a single center and considered a small, though homogeneous, population. However, the current study relies on the well-documented experimental and clinical evidence documenting the importance of adaptive immunity to limit cancer progression.

Lymphocyte subsets were not available as they are not routinely scheduled in our protocol of evaluating and monitoring the patients’ time course.

The knowledge of lymphocyte subsets may better identify subjects whose lymphopenia is prevalently age- or disease-related. Indeed, from an immunologic point of view, aging is characterized by quantitative and qualitative alterations of both adaptive and innate immune cells [45]. The current study cannot distinguish whether lymphopenia was related prevalently to age rather than PC. We postulate that in the study patients Lymphopenia could be prevalently due to the disease as we did not observe different severity of lymphopenia between patients with < 65 years and those with >65 years. Regardless, the relationship between cancer and immunity should always take into consideration the age-related alterations of immunity.

Patients’ nutrition intakes were lacking, so we were unable to consider the relationship between nutrients and immune system cells.

### 4.5. Generation of Work Hypotheses

The current study generates some work hypotheses regarding the possibility of improving L% in lymphopenic (L < 29.7%) subjects with PC:

(1) Does nutrition improve L%? If so, what types of nutrient intakes?
(2) Does the improvement of skeletal muscle mass—the main body protein/amino acid store—support lymphocyte proliferation? Lymphocytes are avid consumers of amino acids for exerting their metabolic activities [29];
(3) May plasma total and/or individual amino acid levels influence lymphocyte proliferation?
(4) Do micronutrients shape the immune response? [46].
(5) May an anti-inflammatory diet contribute to enhancing the prevalence of adaptive immunity? [47].
(6) Is there a BW value(s) (or BMI) at which an interruption of its association with plasma L% may occur?

## 5. Conclusions

The current study shows that peripheral blood L ≥ 29.7% TWBC predicted both chemotolerance and clinical outcomes of patients with advanced pancreatic cancer and highlighted the positive association of circulating lymphocyte percentage with body weight.

## Figures and Tables

**Table 1:** Patient demographics and clinical characteristics.

Variable	n° Patients
Median Age years	
<65	7
66 ≤ x ≤ 79	30
>80	6
Sex	
Female	14
Male	28
Body mass index (kg/m^2^)	
<18.49	7
18.5 ≤ x ≤ 24.99	26
25 ≤ x ≤ 29.99	5
>30	3
Absolute average weight (kg)	61.83 ± 14.4
Stages of Performance Status World Health Organization	
0	28
1	11
2	3
3	0
Previous surgery	
Yes	14
No	28
Previous adjuvant/neoadjuvant chemotherapy	
Yes	5/11
No	27
First-line chemotherapy	
Abraxane-Gemcitabine	32
Gemcitabine	3
FOLFOX6	5
FOLFIRINOX	2
Chemotolerance	
Yes	28
No	14
Reason for discontinuation of therapy	
progressive disease	19
unacceptable toxicity without—progressive disease	9
other	14
Second-line chemotherapy	
Yes	26
No	16
Patient clinical outcomes	
Response to first-line chemotherapy	
Complete response (CR)	0
Partial response (PR)	18
Stable disease (SD)	5
Progressive disease (PD)	19
Progression-free survival	4
Overall survival	9.5

**Table 2:** Patient biohumoral variables and number of chemotherapy cycles tolerated (performed).

Variable		Mean ± SD	Min–Max
Alkaline phosphatase	(nv 40–150 mU/mL)	203.60 ± 167.89	47–800
Alanine transaminase	(nv 11–34 mU/mL)	92.41 ± 145.65	9–716
Amylase total	(nv 25–125 mU/mL)	92.43 ± 117.15	23–480
Aspartate transaminase	(nv 40–150 mU/mL)	51.82 ± 68.15	12–359
Bilirubin total	(nv 0.2–1.1 mg/dL)	4.65 ± 9.86	0.28–55
Calcium	(nv 8.6–10.3 mg/dL)	9.40 ± 0.51	8.5–10.5
Creatinine	(nv 0.55–1.02 mg/dL)	0.69 ± 0.14	0.39–0.93
Estimated glomerular filtration rate	(mL/min/1.73 m^2^)	91.87 ± 12.12	58.47–112.47
Gamma glutamyl transpeptidase	(nv 11–53 mU/mL)	349.58 ± 497.93	9–2111
Glucose	(nv 76–110 mg/dL)	134.79 ± 53.33	52–297
Lactate dehydrogenase	(nv 125–220 mU/mL)	224.50 ± 51.06	166–324
Potassium	(nv 3.50–5.30 mEq/L)	3.79 ± 0.45	2.66–4.66
Sodium	(nv 135–153 mEq/L)	139.17 ± 3.39	132–144
Urea	(nv 10–50 mg/dL)	30.83 ± 7.12	13–42
Hemoglobin	(nv 11.7–15.5 g/dL)	12.51 ± 1.59	7.7–15.6
Erythrocytes	(nv 3.80–5.20 × 10^6^/µL)	4.23 ± 0.60	2.86–5.53
Hematocrit	(nv 35–45%)	37.17 ± 5.07	23.1–46.2
Mean corpuscular volume	(nv 82–98 fl)	88.16 ± 7.03	63.4–99.4
Mean cell hemoglobin	(nv 27–32 pg)	29.78 ± 3.30	20.5–41.3
Mean cell hemoglobin concentration	(nv 32–37 g/dL)	33.76 ± 2.50	30.1–47.8
Red Cell Distribution Width	(nv 11.6–16%)	14.86 ± 1.53	12.9–20.3
White blood cells	(nv 4–10 × 10^3^/µL)	6.71 ± 2.26	2.2–13.9
Neutrophils	(nv 2–8 × 10^3^/µL)	4.60 ± 1.93	1–11.1
Neutrophils%	(%)	67.86 ± 9.45	47.2–84.9
Lymphocytes	(nv 1.5–47 × 10^3^/µL)	1.39 ± 0.58	0.4–3.4
Lymphocytes%	(%)	22.16 ± 8.99	6.8–45.7
Neutrophils/Lymphocytes ratio		3.96 ± 2.74	0.98–12.33
Monocytes	(nv 0.1–1 × 10^3^/µL)	0.54 ± 0.30	0.05–1.7
Monocytes%	(%)	8.07 ± 3.45	0.89–19.52
Eosinophils	(nv 0.1–0.5 × 10^3^/µL)	0.13 ± 0.11	0–0.4
Eosinophils%	(%)	1.96 ± 1.54	0–5.70
Basophils	(nv 0–0.2 × 10^3^/µL)	0.04 ± 0.04	0–0.12
Basophils%	(%)	0.49 ± 0.43	0–2.19
Platelets	(nv 150–450 × 10^3^/µL)	239.46 ± 90.52	81–508
Prothrombin	(nv 70–120%)	94.40 ± 21.88	18–130
International normalized ratio	(nv 0.90–120)	1.13 ± 0.57	0.87–3.77
Carbohydrate antigen 19-9	(<37 IU/mL)	15,757.86 ± 35,367.33	2.5–140,000
Carcinoembryonic Antigen	(no smoking:<2.5; smoking <5 ng/mL)	60.48 ± 216.66	1–1095
n° of tolerated chemotherapy cycles		5.73 ± 3.32	1–18

**Table 3:** Values of Neutrophils (absolute and percentage), lymphocytes (absolute and percentage), neutrophils lymphocytes ratio and number of chemotherapy cycles. (**a**) Lymphocytes ≥ or < 1500 (absolute value); (**b**) lymphocytes ≥ or < 22% (percentage value); (**c**) lymphocytes ≥ or < 29.7% (percentage value); (**d**) response to first-line chemotherapy: PR partial response; SD stable disease; DP disease progression. Data are reported as mean ± SD.

(**a**)							
	**Lymphocytes ≥ 1500**			**Lymphocytes < 1500**			***p***
Neutrophils (N × 1000 × mm^−3^)	5.00 ± 1.88			4.29 ± 1.95			0.14
Neutrophils%	64.68 ± 7.05			70.32 ± 10.45			0.037
Lymphocytes (N × 1000 × mm^−3^)	1.90 ± 0.45			0.99 ± 0.28			<0.0001
Lymphocytes%	25.82 ± 7.85			19.34 ± 8.96			0.017
Neutrophils/Lymphocytes	2.80 ± 1.44			4.86 ± 3.17			0.017
Chemotherapy cycles	5.39 ± 3.16			6.00 ± 3.49			0.53
(**b**)							
	**Lymphocytes ≥ 22%**			**Lymphocytes < 22%**			***p***
Neutrophils (N × 1000 × mm^−3^)	3.58 ± 1.40			5.47 ± 1.91			0.006
Neutrophils%	60.81 ± 6.83			73.91 ± 6.85			<0.0001
Lymphocytes (N × 1000 × mm^−3^)	1.74 ± 0.61			1.09 ± 0.36			0.00038
Lymphocytes%	30.02 ± 5.89			15.43 ± 4.59			<0.0001
Neutrophils/Lymphocytes	2.06 ± 0.57			5.59 ± 2.81			<0.0001
Chemotherapy cycles	6.78 ± 4.15			4.86 ± 2.37			0.15
(**c**)							
	**Lymphocytes ≥ 29.7%**			**Lymphocytes < 29.7%**			***p***
Neutrophils (N × 1000 × mm^−3^)	3.00 ± 1.50			5.15 ± 1.76			0.003
Neutrophils%	58.19 ± 7.46			71.20 ± 7.65			0.00032
Lymphocytes (N × 1000 × mm^−3^)	1.78 ± 0.78			1.25 ± 0.43			0.038
Lymphocytes%	34.03 ± 4.82			18.07 ± 5.90			<0.0001
Neutrophils/Lymphocytes	1.65 ± 0.36			4.76 ± 2.75			<0.0001
Chemotherapy cycles	9.20 ± 3.91			4.55 ± 2.26			0.0005
(**d**)							
	**PR 18 pts**		**SD 5 pts**		**DP 19 pts**		***p***
Neutrophils (N × 1000 × mm^−3^)	4.48 ± 1.25		3.79 ± 1.5		4.9 ± 1.0		0.35
Neutrophils%	66.3 ± 4.5		68.7 ± 2.9		68.8 ± 3.7		0.49
Lymphocytes (N × 1000 × mm^−3^)	1.40 ± 0.51		1.36 ± 0.46		1.35 ± 0.56		0.53
Lymphocytes%	22.17 ± 2.10 ^^^		27.4 ± 3.50 *		19.68 ± 1.63		* *p* < 0.001 vs. PR, DP
							^ *p* < 0.02 vs. DP
Neutrophils/Lymphocytes	3.20 ± 1.68		2.81 ± 0.85 ^^,^°		3.62 ± 1.998		^ *p* < 0.02 vs. DP
							° *p* < 0.05 vs. PR
Chemotherapy cycles	5.7 ± 2.8		8.95 ± 3.2 *		4.78 ± 1.4		* *p* < 0.001 vs. PR, DP

*p* = levels of statistical analysis. * *p* < 0.001; ^ *p* < 0.02; ° *p* < 0.05.

## Data Availability

The data that support the findings of this study are available from the corresponding author upon reasonable request.
